# The Genetic and Molecular Basis for Canine Models of Human Leukemia and Lymphoma

**DOI:** 10.3389/fonc.2020.00023

**Published:** 2020-01-24

**Authors:** Anne C. Avery

**Affiliations:** Department of Microbiology, Immunology and Pathology, College of Veterinary Medicine and Biomedical Science, Colorado State University, Fort Collins, CO, United States

**Keywords:** diffuse large B cell lymphoma, chronic lymphocytic leukemia, peripheral T cell lymphoma, acute myeloid leukemia, acute lymphoid leukemia, dog, canine

## Abstract

Emerging details of the gene expression and mutational features of canine lymphoma and leukemia demonstrate areas of similarities and differences between disease subsets in the humans and dogs. Many features of canine diffuse large B-cell lymphoma resemble the ABC form of human DLBCL, including constitutive activation of the NF-kB pathway, and almost universal presence of double expressing MYC/BCL2 lymphomas. Frequent *TRAF3* mutations and absence of BCL6 expression are differences with the human disease that need further exploration. Canine peripheral T-cell lymphoma is more common in dogs than in people and behaves in a similarly aggressive manner. Common features of canine and human PTCL include activation of the PI3 kinase pathways, loss of PTEN, and the tumor suppressor CDKN2. There is insufficient data available yet to determine if canine PTCL exhibits the GATA3-TBX21 dichotomy seen in people. Common to all forms of canine lymphoproliferative disease are breed-specific predilections for subsets of disease. This is particularly striking in PTCL, with the Boxer breed being dramatically overrepresented. Breed-specific diseases provide an opportunity for uncovering genetic and environmental risk factors that can aid early diagnosis and prevention.

## Introduction

Hematopoietic malignancies are a complex group of disorders that are commonly diagnosed in dogs. When faced with the diagnosis of any kind of cancer in their pet, an owner's decision to treat is based on a myriad of personal and financial factors, but lymphomas are among the most frequently diagnosed and treated types of cancer with chronic lymphocytic leukemia, acute myeloid leukemia, and acute lymphoid leukemia less commonly observed. Chemotherapy protocols, primarily CHOP-based (cyclophosphamide, doxorubricin, vincristine, prednisone), are the current standard of care for aggressive malignancies, and are standardized in the veterinary oncology community. Many dog owners are motivated to participate in clinical trials to help their pet. Thus, dogs can be a useful pre-clinical model for human hematopoietic neoplasia. Discovery of new therapies and refinement of existing ones have the potential to benefit both species. Given the enormous heterogeneity of these types of cancers, however, the utility of the dog as a model will be strengthened by identifying those diseases in each species which share common cell of origin, gene expression profile and driver mutations.

The terms “lymphoma” and “leukemia” encompass an enormous variety of cancers, derived from a broad range of lymphocytes, and myeloid cells from different stages of development, with different functions. The most recent iteration of the WHO classification system for human hematopoietic malignancies uses a combination of histology, epidemiology, immunophenotyping, chromosomal aberrations, mutational analysis, and gene expression profiling to identify different subtypes (1, 2). Discovering the normal cellular counterpart of these tumors drives understanding of the pathogenesis, development of therapies, and preventative measures.

The value of the canine model has been the subject of several recent reviews (3, 4) and generally lies in four areas: a pre-clinical model that has a shortened clinical course compared to human patients, fewer regulatory hurdles to experimental therapies and repeated sampling, the presence of a shared environment, and a degree of in-breeding which has created remarkable breed-specific patterns of disease for the discovery of genetic risk factors.

The goal of this review is to examine hematopoietic malignancies in dogs that may have a human counterpart, and discuss the clinical and molecular basis for their commonalities and differences. The data described here will suggest that, rather than representing an entire WHO subtype, canine tumors may be more similar to a discrete subset of malignancies within one sub-classification. For example, human diffuse large B cell lymphoma (DLBCL) is a heterogeneous disease with an expanding range of subtypes including GC (germinal center) and ABC (activated B cell) forms. DLBCL in dogs appears to be most similar to the ABC form, rather than covering the entire spectrum of disease as seen in people.

## General Approach to the Diagnosis and Treatment of Canine Hematopoietic Malignancy

A consortium of veterinary pathologists applied the WHO classification system to canine lymphoma (5) utilizing immunophenotype and histologic appearance. The comparative features of canine and human lymphoid neoplasms are the subject of a recent review (6). Diagnosis generally begins with analysis of a fine needle aspirate by a clinical pathologist. Often this can lead treatment without further characterization, but more commonly fine needle aspiration is followed by histology (± immunohistochemistry), flow cytometry, or immunocytochemistry. Histology with immunohistochemistry (IHC) can provide a WHO subtype in most cases. Flow cytometry can be used to subclassify most forms of T cell lymphoma (7, 8) but not different forms of B cell lymphoma. Clonality testing is also widely used for those cases where it is difficult to distinguish a reactive from a neoplastic process (9). Although further characterization of canine hematopoietic neoplasms using gene expression, mutational landscape, and chromosomal aberrations are active areas of investigation in many laboratories, these methods have yet to reach routine diagnostic utility. Bone marrow evaluation is often not included in the initial diagnostic workup with the exception of some cases of acute leukemia (see below).

## Types of Canine Lymphoproliferative Disorders

**Figure 1:**
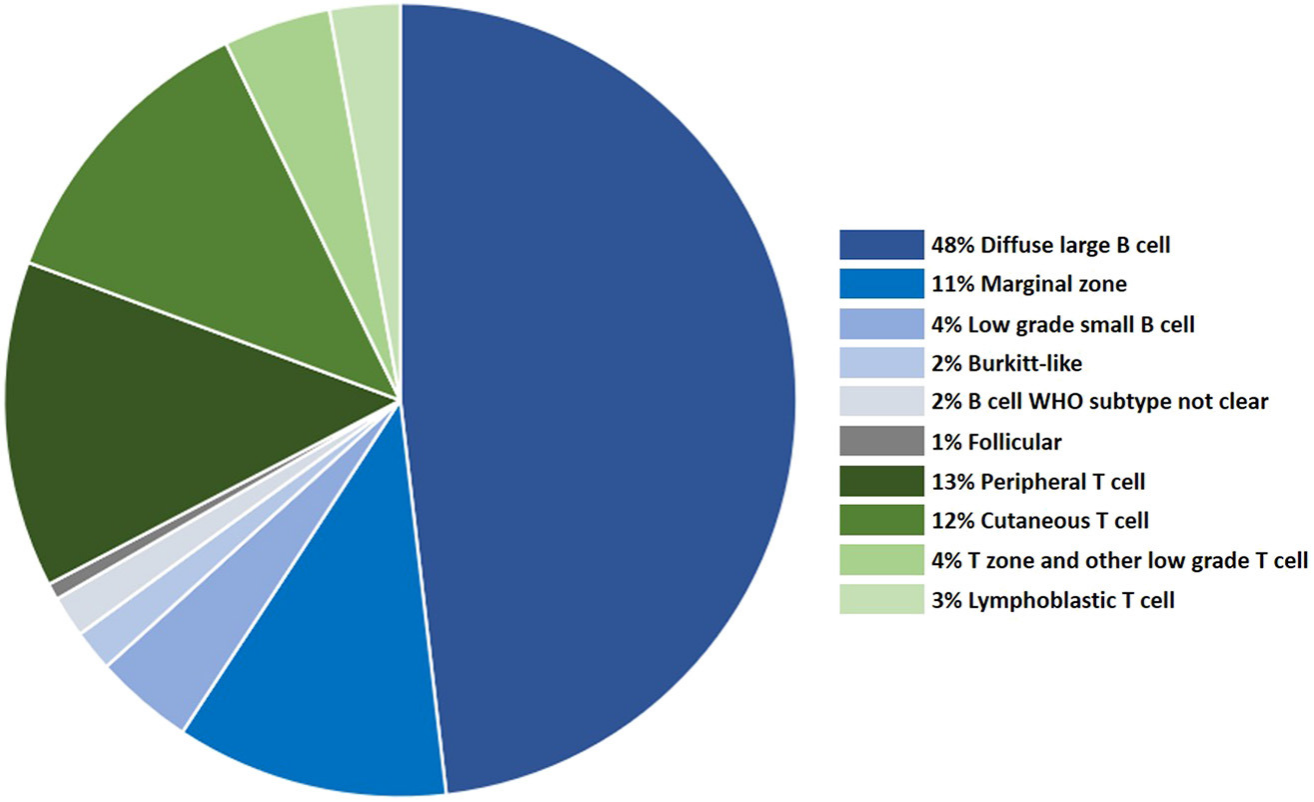
The distribution of canine lymphoma categorized by WHO subtype, based on histology and immunophenotyping.

Although the WHO recognizes more than 60 forms of human lymphoma and leukemia, fewer lymphoma subtypes have been clearly established in the dog [reviewed in Seelig et al. (6)]. WHO subtype is assigned to a canine tumor based on histology and immunohistochemistry (IHC), or for cases of leukemia where the primary disease is in the blood/marrow, by cytologic appearance, and immunophenotyping by flow cytometry. Hodgkin's lymphoma has not been recognized in dogs. Although the frequency of acute leukemias and CLL have not been estimated in dogs, a detailed analysis of over 600 lymphomas categorized by WHO subtype (10) is shown in Figure 1, and this data is supported by one subsequent large study (11).

Precursor neoplasms, mature B cell tumors and mature T cell tumors are all recognized in the dog. Diffuse large B cell lymphoma (DLBCL) is the most common B cell neoplasm in both people and dogs, and peripheral T cell lymphoma the most common T cell neoplasm (10, 11). The frequency of other types differs substantially between breeds, and some tumor types are unique to each. Malignancies derived from human-specific viral infections, such as adult T cell leukemia/lymphoma and Epstein-Barr virus associated tumors do not have common counterparts in dogs. Tumors which appear to be driven by specific translocations, such as follicular lymphoma are rare in dogs, most likely because chromosomal structure differs between species, resulting in barriers to translocation (12). Treatment-associated lymphomas are also not seen in dogs. By contrast, some common canine lymphoid tumors, such as T zone lymphoma (7, 13) and CD8+ T cell leukemias (14), are seen infrequently in people.

## Precursor Neoplasms: Acute Leukemia

### Diagnosis

Canine acute leukemias of lymphoid and myeloid lineage are identified by their clinical presentation, cytologic appearance, and immunophenotype. The diagnosis is most commonly made on peripheral blood. With the exception of rare plasma cell tumors (15) and some cases of B cell lymphoma (16), the expression of CD34 on circulating cells is considered diagnostic for acute myeloid and lymphoid leukemia, but absence of CD34 expression does not rule out the disease. Expression of TdT would be evidence of a precursor neoplasm, but antibodies recognizing canine TdT are not available. Bone marrow evaluation is not common if the diagnosis can be made using peripheral blood, but would be performed if there are peripheral cytopenias and to determine the blast count in acute myeloid leukemia.

### Epidemiology

Epidemiologically, both acute myeloid leukemia (AML) and acute lymphoid leukemia (ALL) appear to be diseases of middle aged dogs; in four separate studies of provisionally diagnosed AML and ALL including over 100 dogs, the median age was reported to be between 7 and 8 years (17–19), and a male predominance was indicated in two studies (17, 19). Both Golden retrievers and German shepherds appear to be frequently affected. It is possible that this observation reflects the popularity of these breeds, but further investigation of this breed association is warranted. If these two breeds are truly predisposed, they would be useful populations for studying underlying genetic risk factors.

### Lineage Determination and Outcome

Once the diagnosis of acute leukemia is established, differentiating AML from ALL is not always straightforward. A combination of morphology, expression of myeloid-lineage proteins (CD11b, CD11c, CD14) and cytochemical analysis (20, 21) is used to diagnose AML. Morphology and lack of myeloid features or antigens is generally used to diagnose ALL, with intracellular expression of CD3 and CD20/Pax5/CD79a used for further lineage assignment. A significant proportion of cases of potential ALL, however, do not express any lineage-defining antigens, and are considered unclassified.

In the author's opinion, canine acute lymphoid leukemias are almost always T cell in origin. Several pieces of evidence support this conclusion: (1) The tissue equivalent (lymphoblastic B cell lymphoma) is almost never diagnosed by histopathology, whereas lymphoblastic T cell lymphoma, while not common, has been identified in several studies (5, 10), (2) When examining copy number variation in canine ALL, recurrent losses in the T cell receptor α/δ, and T cell receptor β region were observed, consistent with T cell receptor rearrangements, but similar losses were not identified in immunoglobulin heavy or light chain regions (22), (3) Evidence for B cell lineage in reports describing B-cell ALL have relied entirely on the expression of CD79a which does not have high lineage fidelity (23, 24). Despite these observations, further proof of this hypothesis will require additional immunophenotypic and gene expression studies.

Regardless of lineage, outcomes in acute leukemia are dismal, even with aggressive chemotherapy (14, 17–19). Median survivals are reported to be 25–50 days.

### Mutational Analysis

There are limited studies of the molecular features of acute leukemia and it is too early to determine if canine ALL and AML will be appropriate models for their human counterparts. Some commonalities, however, have been described. *FLT3, NPM, DNMT3A, RAS*, and *KIT* are among the most frequently mutated genes in human AML (25, 26) and FLT3, RAS, and KIT are potential therapeutic targets. In two studies that encompassed a total of 43 dogs with AML or ALL, a FLT3 internal tandem duplication (ITD) was found 5 cases (12%) (27, 28). Because of the difficulty in lineage assignment, it is unclear if these cases were myeloid or lymphoid. Treatment with a FLT3 inhibitor resulted in marginal decreases in the growth characteristics of a canine cell line containing the ITD and inhibition of downstream signaling pathways (28). In the same study (27), 25% of AMLs were noted to have *NRAS* missense mutations and 20% had *KIT* mutations. In a separate investigation of the DNA methylation status of AMLs, *DNMT3A* mutations were not identified (29).

Taken together the data suggest that canine acute leukemias, in particular AMLs, may share some common mutations with human AML, and with further characterization of the canine disease may provide a useful model for testing new therapies. The very poor outcomes currently seen in canine acute leukemia mean that such testing would benefit both dogs and humans, and owners would be eager to try new therapies that could promise better results.

## Mature B Cell Neoplasms

### Diffuse Large B Cell Lymphoma

#### Diagnosis

Diffuse large B cell lymphoma (cDLBCL) is the most common subtype recognized in dogs and people. In dogs, the histologic diagnosis relies on recognition of a diffuse pattern with uniformly large nuclei (5). Pax5, CD79a, and CD20 (cytoplasmic) are all used to indicate B cell lineage by IHC. Immunophenotyping can also be performed by flow cytometry. Antibodies to cell surface CD19 and CD20 are not available for dogs, but anti-CD21 and anti-CD22 reliably identify B-cell lineage tumors, and intracellular staining for CD79a can also be used but is less specific. Although the lineage (B or T cell) can be determined by flow cytometry, different forms of B cell lymphomas (BCL) cannot be identified using this method.

#### Epidemiology

cDLBCL affects dogs over a wide range of ages, with the median age of 7–9 years (10, 30, 31). It is not clear if there is a breed-specific predilection for this disease, but several studies of histologically confirmed cDLBCL indicate Golden retrievers, Labrador retrievers, Bernese mountain dogs and German shepherds are commonly affected breeds (10, 30, 31). Dogs are typically treated with CHOP, with overall survival times varying between studies, from 300 to 500 days (31–33). Although a number of clinical trials of anti-CD20 therapy in dogs with B cell lymphoma are in progress, to date clinically efficacious antibody therapy is not available for dogs with BCL.

#### Gene Expression Profiling and Mutation Status

Gene expression profiling has demonstrated that the majority of human DLBCL (hDLBCL) arise from cells within the germinal center (GC DBLCL), or from cells immediately post-germinal center differentiating toward plasma cells (activated B cell or ABC DBLCL) (34). Although histologically indistinguishable, these forms of DLBCL most likely represent molecularly distinct entities at different points along the B cell differentiation pathway, because mutations, large scale chromosomal aberrations and the pathways that drive their proliferation differ between the groups (35, 36). Notably, activation of the NF-kB/B cell receptor signaling pathways are characteristic of ABC DLBCL, but not GC DLBCL [reviewed in Young et al. (37)].

Global gene expression profiling of cDLBCL does not precisely parallel either subtype, but appears to have more in common with human ABC DLBCL. Several lines of evidence support this idea. Gene expression profiling from cDLBCL compared with normal lymph nodes shows an enrichment for genes in the B cell receptor signaling pathway (38, 39) and the NF-kB signaling pathway (38–40). These observations were corroborated by biochemical studies demonstrating that 24/24 dogs with cDLBCL exhibited constitutive activation of the canonical NF-kB pathway (the non-canonical pathway was not investigated in this study) (41, 42). The expression of downstream anti-apoptotic genes (*BCL2, c-FLIP*, and *XIAP*) was increased in the majority of these cases. Inhibition of the upstream IKK complex using a NEMO binding domain peptide inhibited phosphorylation of IkB and increased apoptosis *in vitro* (41), and showed some degree of efficacy in these same assays when administered intra-nodally or intravenously (41, 42).

To some extent, analysis of mutations harbored by cDLBCL also point to activation of the NF-kB pathway, but with some confounding observations. Three studies have investigated mutational features of canine BCL—either specifically subtyped cDLBCL, or pooled cases of BCL which were most likely dominated by cDLBCL. Together they included 129 total dogs (43–45). Notably, 20–30% of these lymphomas had inhibitory mutations in *TRAF3*. TRAF3 is a negative regulator of the non-canonical NF-kB signaling pathway, and when inactivated leads to constitutive processing of p100 to p52 for translocation into the nucleus with its partner, RelB (46, 47). In contrast to signals through the B cell receptor and toll like receptors (through MyD88), the non-canonical NF-kB pathway is activated in response to differentiation signals such as those transmitted through CD40 and BAFF. Although *TRAF3* mutations in hDLBCL were rare (36) or not found at all (43) in both studies a subset of hDLBCL, 9% of cases exhibited chromosomal loss in the region containing *TRAF3*, a finding further supported by Green et al. (48). ABC DLBCL had the most frequent losses (36).

Other mutations that promote constitutive NF-kB activation associated with the canonical NF-kB pathway were found in cDLBCL at low levels in only one study: *TNFAIP3* (14% of 14 cases) and CD79b (7% of 14 cases) (43). Thus the overall implication of the available mutational analyses is consistent with the idea that the NF-kB pathway is important to cDLBCL.

Further support for the idea that cDLBCL more closely resembles the ABC form is the observation that virtually all cDLBCL are “double expressers”—a very high proportion of cells in canine DLBCL express both MYC and BCL2 (49), a feature which is associated with significantly poorer outcomes in people, and more frequently seen in ABC DLBCL (although can be present in all forms) (50). A parallel finding is the enrichment for genes in the MYC pathway in cDLBCL (39). High expression of MYC and BCL2 in some human cases is the result of translocations which free the genes from transcriptional constraints, but not all cases with high levels of protein expression have the translocation. *MYC-IGH* translocations have been observed in the dog, but only in Burkitt's lymphoma (51). Dog cancers of all types, including lymphomas, frequently have gain of part or all of chromosome 13, which carries *MYC* and *KIT* (52, 53). This may be the most common reason for the high level of *MYC* expression.

#### Differences Between Canine and Human DLBCL

Despite the accumulating data on similarities between cDLBCL and human ABC DLBCL, there are some significant differences that need to be clarified in order to fully exploit the dog as a pre-clinical model. Perhaps most importantly, cDLBCL appears to almost never express the BCL6 protein (1 positive case of 59 total tested) (38, 54). BCL6 is readily detectable in germinal centers of normal canine B cells by IHC (54). BCL6 is a transcriptional repressor that is key to the germinal center reaction. One of its major roles is to inhibit terminal differentiation of B cells to plasma cells or memory cells, thereby maintaining cells within the germinal center cycling between light and dark zones to undergo proliferation, somatic hypermutation and selection. *BCL6* is downregulated when cells exit the germinal center.

While ABC DLBCL are more frequently BCL6 negative than GC DLBCL (55), the lack of any evidence of BCL6 expression in dogs is unusual. Furthermore, while upregulation of IRF4 (Mum1) is a feature of post-germinal center B cells that have down-regulated BCL6, and seen more frequently in ABC DLBCL (34), IRF4 does not appear to be expressed in most cDLBCL when assessed using IHC (38).

Overall, the evidence suggests that cDCBCL is dissimilar to GC DCLBL. In addition to the data described above, mutation analysis did not identify genes that are commonly found in GC DLBCL such as *KMT2D, MYD88, CARD11, EZH2*, and *CREBBP* (36, 56, 57). Given that the ABC subtype of DLBCL is most closely associated with activation of the NF-kB pathway, the combined findings of mutation analysis, gene expression profiling and IHC suggest that canine DLBCL is more similar to the ABC form that GC form.

#### Immunoglobulin Heavy Chain Gene Mutation Status

B cells that have been through the germinal center have hypermutated immunoglobulin variable region genes (IGHV) as a result of affinity maturation and selection and presence of hypermutated IGHV genes indicates a B cell has (in most cases) traveled through the germinal center. To determine mutation status in B cell neoplasms, IGHV gene sequences from the tumor are compared to unmutated sequences—the latter data taken from compiled germline sequences from a large number of individuals (http://www.imgt.org). In people, IGHV genes are considered to have undergone hypermutation if there is <98% sequence similarity compared to germline counterpart (58). The analysis of IGHV mutation status in dogs is complicated by the fact that there is only one germline sequence available for each of the 80+ IGHV genes. This allele is from the Boxer originally sequenced for the canine genome (59). Because only one allele for each V gene has been sequenced, the degree of allelic variation in IGHV gene is unknown in the dog. When comparing the sequence of a neoplastic IGHV to the available germline, differences may be due to either hypermutation or due to allelic variation.

With this caveat in mind, IGHV genes in 52/52 cases of canine B cell lymphoma from various breeds (29 definitively established to be cDLBCL) had >98% identity with their germline counterpart from the Boxer (60), indicating, as expected, that they were derived from germinal center or post germinal center B cells. A separate study, which examined only Boxer large cell B cell lymphomas (not further classified by histology) found that only 64% exhibited hypermutation (61). Thus, while it is likely that most cDLBCL have evidence of being derived from germinal center B cells based on IGHV mutation status, a precise enumeration IGHV mutation status in canine B cell lymphoma will depend on a larger database of IGHV alleles.

#### Summary

Although much work needs to be done, the available evidence suggests that cDLBCL is most similar to ABC DLBCL. Gene expression, biochemical data and mutation analysis point to a role for the NF-kB signaling pathway, which is central to ABC DLBCL. Co-expression of high levels of BCL2 and MYC is seen in virtually all cDLBCL, a feature more common to the ABC subtype. cDLBCL has some unique characteristics as well, including the possibility that the non-canonical NF-kB pathway may be prevalent in some cases, and the observation that BCL6 is not expressed. As noted for acute leukemias and T cell lymphoma, it is likely that cDLBCL will be analogous to discreet subsets of hDLBLC, rather than reflecting the entire range of subtypes in this diverse disease.

### Other Mature B Cell Neoplasms

Follicular lymphoma and mantle cell lymphoma have been described in dogs, but are uncommon, and little information beyond descriptive data is available. Some information is available for two more common B cell tumors, marginal zone and B-cell CLL.

#### Marginal Zone Lymphoma

Canine marginal zone lymphoma (MZL) is defined by a nodular pattern and intermediate-sized cells with a central nucleolus. This disease can be found in both the spleen and lymph nodes. While originally considered to be a single disease, studies have shown that while splenic marginal zone lymphoma has good long-term survival with splenectomy alone (62), nodal marginal zone lymphoma is as aggressive as cDLBCL (63). In later stages of the disease, pathologists can have difficulty distinguishing DLBCL from NMZL, often referring to NMZL as “late NMZL” to reflect the fact that the tumor contains marginal zone-like cells, but the characteristic nodular pattern is lost (38). Molecular characterization of this disease is lacking, other than the observation that gene expression profiling was unable to distinguish histologically diagnosed cDLBCL from nodal marginal zone lymphoma in three separate analyses (38, 64, 65).

#### B Cell CLL

B cell chronic lymphocytic leukemia (B-CLL) in dogs is diagnosed by expansion of small, mature-appearing B cells in the blood, and an indolent clinical course (14). Unlike the human disease, canine B-CLL cells do not express CD5. It is seen primarily in small breed, older dogs (66). Strikingly, despite the prevalence of large breed dogs such as German shepherds and Golden retrievers in the population of cDLBLC, these breeds virtually never develop B-CLL (66). Small breed dogs tend to have longer lifespans than large breeds, but this not the entire reason for the observation. Golden retrievers are the most common breed to develop indolent T zone lymphoma, which is also a disease of older dogs (median age at presentation 10 years for both diseases) (67). Thus, the intriguing breed-specific resistance to B-CLL most likely has a more complex explanation than age.

No comprehensive gene expression or mutational analysis has been performed for canine B-CLL, but analysis of copy number variation highlighted a region of shared aberration with the human disease (22). Twelve percent of canine B-CLL cases exhibited loss of a chromosomal region encoding miR-15a/miR16-1—loss of this region is present in approximately 55% of human patients [reviewed in Spina et al. (68)]. These microRNAs are thought to control expression of *BCL2*, and loss of expression results in *BCL2* upregulation. A variety of other shared regions of gain and loss between human and canine B-CLL were observed, but all await further confirmation by gene expression and functional studies.

One of the most important prognostic factors in human B-CLL is the mutation status of the IGVH gene (69). Patients with unmutated IGHV genes tend to have more aggressive disease. We have started to explore the role of IGHV mutation status in canine B-CLL, and found that the majority of cases exhibit IGHV mutation, with one exception. B-CLL in the Boxer breed carry unmutated IGHV genes (61), but mutation status and outcome has not yet been linked in individual dogs. Nonetheless, with further confirmation and gene expression studies, which are currently underway, the Boxer breed may be a useful model for aggressive B-CLL.

## Mature T Cell Neoplasms

### Peripheral T Cell Lymphoma

The frequency of peripheral T cell lymphoma in dogs, and early indications of genetic similarities with the human disease, perhaps offers the most fruitful avenue for comparative investigation between the two species. The two most frequent forms of nodal T cell lymphoma in dogs are peripheral T cell lymphoma not otherwise specified (PTCL-NOS) and T zone lymphoma. The latter is classified as a very rare form of human PTCL in the WHO system, but it is discussed separately from PTCL in veterinary medicine because it is common, and clearly distinct from the other types—dogs are older, the disease is indolent and often not treated, and the defining feature of T zone lymphoma is loss of CD45 expression (7, 70), which is not seen in other canine lymphomas. Anaplastic large cell lymphoma (71) and angioimmunoblastic T cell lymphoma (AITL) are diagnosed rarely if at all in dogs (5).

#### Epidemiology

Canine PTCL-NOS (cPTCL) is most common in the Boxer breed. This breed-specific predilection is striking and has been noted in numerous studies in both the U.S. and Europe (10, 72–74). cPTCL affects middle age dogs (median 7 years) and has a slight male predominance (8, 72). Mediastinal involvement is seen in approximately half of cPTCL cases, and half of all cases are hypercalcemic (these features overlap but also exist independently). Peripheral blood involvement, including the presence of circulating cells and peripheral cytopenias, is very rare. Median survival is approximately 158 days with CHOP or modified CHOP chemotherapy (72, 75, 76).

#### Diagnosis

A study of 73 cases of nodal cPTCL with paired histology and immunophenotyping by flow cytometry demonstrated that cPTCL can be reliably diagnosed by flow cytometry alone (8). cPTCL most commonly expresses CD4, high levels of CD3, low levels of class II MHC and often exhibits loss of CD5 expression, similar to hPTCL. A smaller number of cases express CD8, neither CD4 nor CD8, or both antigens. The cells do not express CD34, and gene expression profiling on a limited number of cases did not identify upregulated *TdT* expression compared to normal CD4 T cells (8). These findings support the classification of these tumors as mature T cell lymphomas, rather than precursor neoplasms despite the frequent mediastinal involvement.

#### Gene Expression Profiling and Mutation Status

Human PTCL-NOS (hPTCL) has been divided by gene expression profiling into two main subtypes—those which are characterized by upregulation of the transcription factor GATA3 and the downstream pathways it controls, and those characterized by upregulation of the transcription factor TBX21 (T-bet), and downstream pathways (77, 78). GATA3 and TBX21 are drivers of Th2 and Th1 helper T cell differentiation, respectively, and the GATA3 subtype is associated with reduced survival. There is some evidence that GATA3-dominant hPTCL affect the tumor microenvironment by driving macrophages toward an alternative, suppressive phenotype (79) which may contribute to poor outcomes.

Gene expression profiling by RNA seq (8), mutational analysis (44, 80), and copy number variation (52, 53) have been described in separate studies of canine T cell lymphoma. There is insufficient gene expression data currently available to determine the extent of heterogeneity of cPTCL, and if this disease can be subdivided into GATA3 and TBX21 associated tumors. However, gene expression studies did provide some information about possible relevant pathways. In an RNA seq study of 6 cPTCL, increased expression of the *MTOR* gene and decreased expression of *PTEN* was observed in all 6 cases when compared with purified normal CD4 T cells (8). Gene set enrichment analysis confirmed the transcriptional programs associated with upregulation of *MTOR* and down-regulation of *PTEN* were consistent with this observation. PTEN is an inhibitor of the phosphatidylinositol 3-kinase (PI3K) pathway, and release of inhibition results in downstream activation of a variety of cell survival pathways (81). In hPTCL, dysregulation of this pathway is seen in PTCL-GATA3 (78) and a clinical trial using a PI3K inhibitor showed some degree of efficacy in hPTCL (82).

Gene expression data is supported to some extent by mutational analysis. Elvers et al. described exome sequencing from 41 cases of canine T cell lymphoma (44). Samples were derived from two breeds, Boxers and Golden retrievers, which exhibited a different array of mutations. These tumors were not subtyped by histology, but the high frequency of T zone lymphoma in Goldens, and the high frequency of PTCL in Boxers suggests the histologic subtypes were probably quite different in these two groups. When focusing on the likely cPTCL mutations (Boxer breed, 16 dogs), this study found that 25% of cases had *PTEN* mutations, consistent with the gene expression data. Targeted sequencing of cancer genes similarly identified *PTEN* mutation in a subset of T cell lymphomas (80).

Another observation from both exome sequencing and analysis of variants in RNA seq data is that 16–25% of canine PTCL have a functional mutation in the SATB1 gene (8, 44). SATB1 is a transcriptional repressor that is predominately found in thymocytes and plays a key role in T-cell development (83). It has variable effects when dysregulated in human cutaneous T cell lymphomas and anaplastic T cell lymphomas (84). It is possible that a subset of cPTCL (particularly those with mediastinal involvement) are derived from a mature, naïve T cell precursor, and *SATB1* mutation may be functionally relevant to this group of tumors.

cPTCL have more extensive chromosomal aberrations than canine B cell lymphomas (53). In addition to frequent gains of chromosomes 13 (containing the *MYC* gene) and 31 (also noted for B cell lymphomas), cPTCL exhibited frequent losses of chromosomes 11, 17, 22, 28, and 38. The CDKN2 gene (p16) is found on canine chromosome 11, and loss of CDKN2 was further validated in a follow up study (85) which found that CDKN2 loss was specific to PTCL and not seen in low grade T cell lymphomas or B cell lymphomas. This finding is particularly notable because CDKN2 is a tumor suppressor: loss of expression was noted in 45% of human PTCL-GATA3 tumors (78) and was associated with a poorer prognosis.

Characterization of canine PTCL is clearly in its early stages. Important questions to be answered included the nature of the driver mutations and the identification of the cell of origin of discreet subsets of disease. As with cDLBCL, dogs may not represent the entire spectrum of human PTCL, but could be used to focus attention on particular subsets of the disease.

## Exploiting Breed-Specific Diseases for Prevention and Disease Prediction

Several instances of breed-specific patterns of disease are noted above. Such observations mean that genetic and environmental risk factors for cancer may be identified in smaller prospective studies, taking less time, than would be necessary in people. The leading cause of death in Golden retrievers is hematopoietic malignancy (86), and in 2012 a prospective study of >3,000 Goldens, enrolled at age 2 or younger, was undertaken (87). The goal of this study was to collect environmental and biological data for correlation with the future diagnoses of malignancy and other diseases. Sadly, in this group of dogs, the oldest of which is 7, 42 cases of lymphoma have already been identified (1.3%) (Diehl, K. personal communication). It will be several years before the prospective data can be compiled to examine risk factors, but this study is an example of how the dog may not only be used to investigate treatments, but to identify possible interventions before cancer gains a foothold.

## Future Directions

The short-term goals that will allow the dog to be deployed as a pre-clinical model for human hematopoietic malignancy include a comprehensive analysis of gene expression, mutational landscape and epigenetic features for each subtype of disease. These goals are readily achievable with the tools currently available to researchers, with some caveats—the canine genome is less well-annotated than the human genome, and a full catalog of population level polymorphisms is still being developed. A greater challenge will be creating more focused diagnostics (small gene expression panels, better antibody-based classification using IHC or flow cytometry) for routine clinical use. The availability of such focused diagnostics will be essential for taking full advantage of the canine model for investigating new therapies, allowing for rapid classification of cases and enrollment in drug trials. An additional benefit of more precise classification of canine hematopoietic malignancies will be the identification of even more breed-specific tendencies allowing for discovery of additional genetic risk factors. While the specific risk genes are unlikely to be identical between species, the pathways or processes affected may be similar. Evaluation of genetic and environmental risk factors, coupled with the ability to test new therapies, can make the dog a comprehensive large animal model for one of the most important cancers affecting both species.

## Author Contributions

AA designed and wrote the review.

### Conflict of Interest

The author declares that the research was conducted in the absence of any commercial or financial relationships that could be construed as a potential conflict of interest.
